# A Novel Nanowire Assembly Process for the Fabrication of CO Sensor

**DOI:** 10.3390/s18041234

**Published:** 2018-04-17

**Authors:** Biyao Cheng, Shuming Yang, Tao Liu, Ali Vazinishayan

**Affiliations:** State Key Laboratory for Manufacturing Systems Engineering, Xi’an Jiaotong University, Xi’an 710049, China; chengbiyao@stu.xjtu.edu.cn (B.C.); liu8483@xjtu.edu.cn (T.L.); Alivazinishayan@gmail.com (A.V.)

**Keywords:** nanowire assembly, combing process, ZnO, CO sensor

## Abstract

Nanowires have been widely studied due to their outstanding mechanical and electrical properties; however, their practical applications are limited to the lack of an effective technique for controlled assembly. In the present work, zinc oxide (ZnO) nanowire arrays were assembled via a combing process using a makeup brush and the nanodevice was fabricated. The current–voltage (I–V) and ultraviolet (UV) characteristics of the device indicate stable and repeatable electrical properties. The carbon monoxide (CO) sensing properties were tested at operating temperatures of 200, 300 and 400 °C. It was found that ZnO based sensor exhibited the highest sensitivity to CO at 300 °C due to the change of dominant oxygen species. Comparing with others result, the sensitivity of the fabricated sensor exhibits higher sensing performance. The sensing mechanism of the CO sensor is also discussed.

## 1. Introduction

Among different kinds of nanomaterials, nanowires have attracted great attention due to unique properties such as anisotropic structures, miniaturized dimensions, high sensitivity and fast response, which suit a variety of applications [1,2,3,4,5,6,7,8,9,10,11,12]. If nanowires could be aligned and arranged into patterns, the impact would be tremendous in many areas, from nanoscale electronics and optoelectronics to molecular sensing. Scalable and controlled assembly of nanowires, however, remains unsolved for their potential integration in nanodevice fabrication. Thus, extra efforts have been made on tackling the controlled assembly, such as using electric-field assisted alignment [13], flow assisted alignment [4], Langmuir–Blodgett (LB) technique [14,15,16,17], contact printing [18,19], super-crystalline self-assembly [20], laser-induced method [21], optical manipulation [22], etc. Smith et al. [13] developed an electric-field assisted assembly technique to position individual Au nanowire suspended in a dielectric medium between two electrodes on the SiO_2_ substrate. However, this method is limited to assembling metallic nanowires. The Langmuir–Blodgett technique is usually used to fabricate nanodevices [23,24] and assemble one=dimensional nanomaterials into large-area. Kim et al. [15] applied the LB technique to assemble single layers of low aspect ratio nanorods into close-packed structures suggestive of liquid crystalline phases. Whang et al. [16] used the LB method to control organization and hierarchy of silicon nanowires in parallel. It should be noted that these techniques require complex surface modification and the alignment of nanomaterials rely on the surface pressure controlling. Fan et al. [19] developed the kind of contact printing method to transfer regular arrays of semiconductor nanowires from the donor to patterned receiver substrates; however, it might scrap the surface due to poor contact between nanowires and the substrate. Other approaches mentioned above need additional post-alignment contact, which cause significant increment in both the cost and the complexity. Therefore, a highly efficient and simple assembly method of nanowires is in urgent need of constructing functional nanodevice.

In this paper, a novel strategy is proposed to assemble ZnO nanowires by a simple combing process. Using this method, ZnO nanowires are well assembled on the silicon substrate and a nanodevice with nanowires assembly is fabricated. Compared with previous methods on fabricating the nanowires-based gas sensor, the approach presented here is preferable, as this simple combing method does not require expensive instruments or complex equipment.

## 2. Experiment

### 2.1. Assembly Mechanism of Nanowires Using the Combing Process

**Figure 1:**
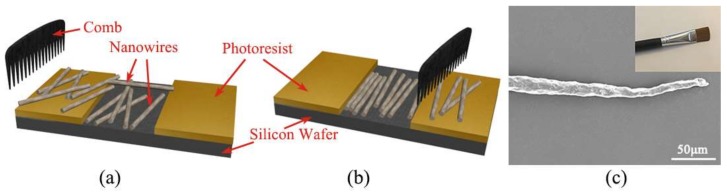
Schematic diagram of the combing process. Nanowires array before (**a**) and after combing (**b**). (**c**) A single brush hair of the makeup brush; the inset shows the makeup brush used in practice.

As schematically shown in Figure 1a, many nanowires are randomly dispersed and placed on the silicon substrate. A groove is fabricated by etching the photoresist. Moving a comb (a common makeup brush) from the left side to the right side can translocate the nanowires under the pressure of the comb teeth. Figure 1b shows some nanowires are trapped inside the groove and forced to be orderly assembled. Meanwhile, the comb swipes away the remaining nanowires outside the groove. The makeup brush and SEM image of a single brush hair with gold coating are shown in Figure 1c. It is observed that the hair is not in regular size with diameter around 10 micrometres at the tip. However, makeup brush is a good comb candidate since the hairs of brush are flexible enough to completely touch the substrate.

### 2.2. Fabrication of ZnO Nanowire Device

Nanostructures from ZnO exhibit interesting electrical characteristics due to their typical n-type conductivity [25] and they can easily absorb ultraviolet (UV) photons efficiently. Hence, a ZnO nanostructure device can be used for sensing purpose [26]. In general, one-dimensional ZnO nanowires can be synthesized by various kinds of techniques. In this work, ZnO nanowires were fabricated using a process described by Hsueh et al. [27].

**Figure 2:**
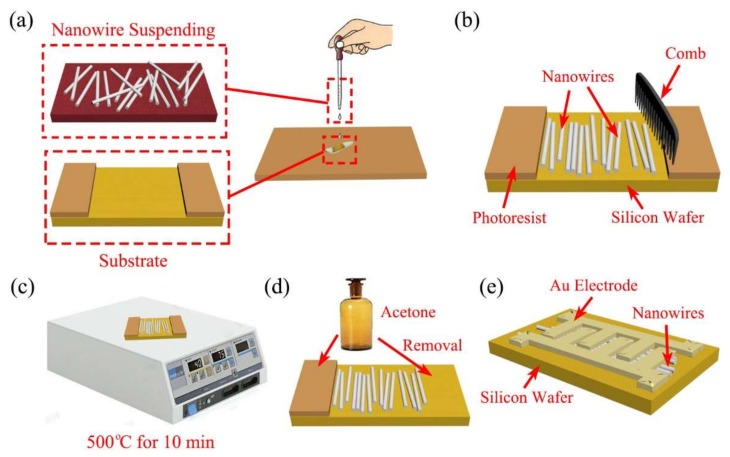
Simplified process flow diagram of the assembly of nanowire on the silicon substrate and nanodevice fabrication. (**a**) Drip a drop of the suspension on the substrate; (**b**) combing process after 24 h; (**c**) bake on hot plate for ~10 min at 500 °C; (**d**) remove the photoresist and not adhered nanowires by acetone; (**e**) standard electron-beam lithography and metal deposition.

To assemble nanowires on the silicon substrate and fabricate the nanodevice, the whole process is performed as depicted in Figure 2. The nanowires were steeped in deionized water, forming suspension with ultrasonic vibration for about 10 min. (a) A drop of the suspension was dripped on the substrate, in which the nanowires were not aligned. (b) Then combing was performed at a constant velocity after air dried naturally for about 24 h. (c) As ZnO nanowire became soft when external temperature reached above 400 °C, the nanodevice was baked on hot plate for ~10 min at 500 °C to make nanowires strongly adhere to the substrate via Van der Waals force. (d) The nanodevice was placed in acetone to remove the photoresist and not adhered nanowires. Likewise, the combed nanowires assemblies could be used as nanodevice to validate their performance. Finger shaped electrodes nanodevice was fabricated, as it is a popular design for electrical device [28], which can increase the contact area between the nanomaterials and electrodes. Hence, (e) Au electrodes were added onto top of nanowire with standard electron-beam lithography and metal deposition.

## 3. Results and Discussion

### 3.1. ZnO Nanowires Assembly and Nanowires Device

**Figure 3:**
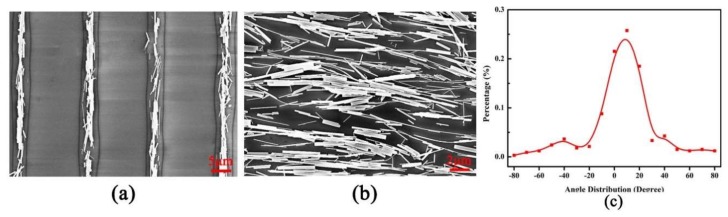
(**a**) FESEM image of four nanowires arrays; (**b**) the zoom-in image of the first column; and (**c**) the angle distribution of ZnO nanowire in the first column.

Field emission scanning electron microscope (FESEM) was used to characterize the nanowire assembly results. Figure 3a shows typical FESEM images of four ZnO nanowires arrays after combing on the silicon substrate. Figure 3b is the zoom-in image of the first column, where all the nanowires are aligned in the same direction. To better understand the effect of combing process, the angle distribution (degree) versus percentage was measured by AutoCAD software, as shown in Figure 3c. It indicates more than 80% of the nanowires are aligned within 20 degrees with respect to the combing direction. From the image analysis, it is concluded that the combing process makes the nanowire well aligned on the substrate.

**Figure 4:**
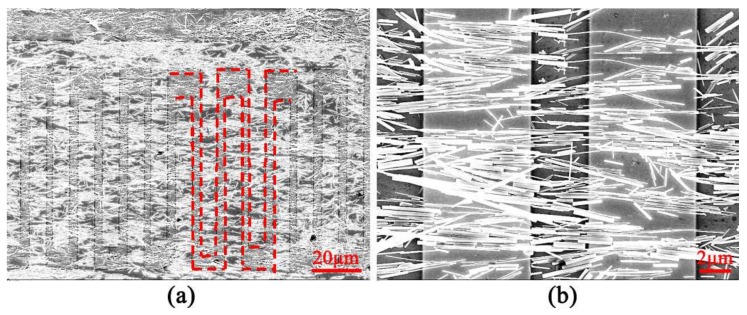
FESEM images of the device: (**a**) ZnO nanowires device with finger shape Au electrodes after the process of nanowire assembly on the Si substrate (red dotted line denotes the finger shape electrodes); and (**b**) the zoom-in image of the device.

Figure 4a displays the FESEM image of ZnO sensor after adding the Au electrodes on the nanowire assembly. Totally, there are 15 electrode pairs with the width of around 3 mm and the length of 80 mm and the gap between two adjacent electrodes is around 2 mm. Interestingly, Figure 4b demonstrates that the ZnO nanowires remain aligned after the nanodevice was fabricated, which demonstrates the stability of ZnO nanowires fabricated with our homebrewed assembly technique.

### 3.2. Characteristics Testing of ZnO Nanowire Device

**Figure 5:**
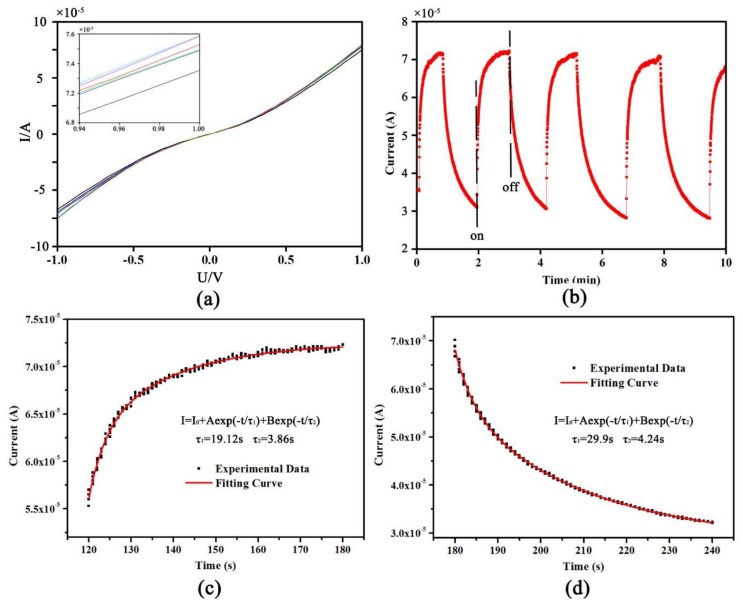
Characteristics testing results of ZnO nanowire device: (**a**) I–V characteristics; (**b**) ultraviolet characteristics at the bias voltage of 0.5 V; (**c**) rising process with the light; and (**d**) the decaying process of the current without the light (the solid curves represent fitting curve based on Equation (3)).

The I–V curve of the ZnO nanodevice is highly nonlinear and nearly symmetrical with respect to the applied voltage, as seen in Figure 5a. These I–V characteristics are typically obtained for Schottky contact and semiconductor grain boundaries [29,30]. These I–V curves are nearly overlapped with six repetitions which imply the current response of the sensor is remarkably stable. The current is about 76 μA under the bias voltage of 1 V, indicating a low resistance of ~13 KΩ. Repeatability and smoothness of the I–V curve demonstrates the stable contact between nanowires and electrodes.

Generally, the carriers inside the ZnO nanowire are excited and jump to high energy level when irradiated by ultraviolet [31]. The UV illumination is used to test the electrical contact between nanowires and electrodes, as the UV sensing phenomenon originates from the alteration of the charge carrier density. In the experiment, the UV source was interrupted periodically to obtain the response curve of the sensor with light “ON” and the recovery curve with the light “OFF”. The response behaviour of the device was characterized by measuring the current under fixed bias of 0.5 V as a function of time when the device was periodically exposed to the UV light, which is shown in Figure 5b. Initial current 3.3 μA increased to 72.2 μA under the UV illumination, which is nearly a 22-fold enhancement in response. The result is higher than the value reported by Oliver et al. [32]. Fundamentally, the photoresponse of ZnO is due to the photon adsorption and desorption of oxygen [33,34]. In the dark, oxygen molecules are absorbed on the surface of nanowire as negatively charged ions by capturing free electrons from the n-type ZnO, leading to a depletion layer with low electrical conductivity near the nanowire surface:

(1) $$O_{2}(g)+e^{-}\to O_{2}^{-}(\mathrm{ads})$$

UV light absorption generates electron–hole pairs and discharge the adsorbed oxygen ions through surface electron–hole recombination:

(2) $$h^{+}+O_{2}^{-}(\mathrm{ads})\to O_{2}(g)$$

This may help to explain why the photoresponse is higher than that in Ref. [32]. The better alignment degree of the nanowires led to the larger contact area between nanowires, and therefore the ability to control the barrier height is enhanced. High performance of our device is associated with the multiple barriers. The UV light induced desorption of oxygen at the boundary changes the barrier height and width as well as improves UV sensitivity. As the depletion layer thickness is related to the oxygen coverage, the depletion layer width changes with absorption–desorption of oxygen. 

Figure 5c,d shows the fitting curves for the rising process with the light on and the decaying process with the light off, respectively. Both the ascending and descending processes can be fitted by the dual exponential curve with the form:

(3) $$I=I_{0}+A\exp(-t/\tau_{1})+B\exp(-t/\tau_{2})$$

with estimated response time of 22.98 s and the decay time of 34.14 s. In Equation (3), *I* is the current, *I*_0_ is the dark current, and *τ*_1_ and *τ*_2_ are the time constant of quick process and slow process, respectively. The quick process corresponds to the relaxation process which is related to the surface state of ZnO nanowires and that slow process corresponds to the relaxation process which is related to the deep level defect of ZnO nanowire. 

### 3.3. Gas sensing Properties of the ZnO Nanowire Assembly

**Figure 6:**
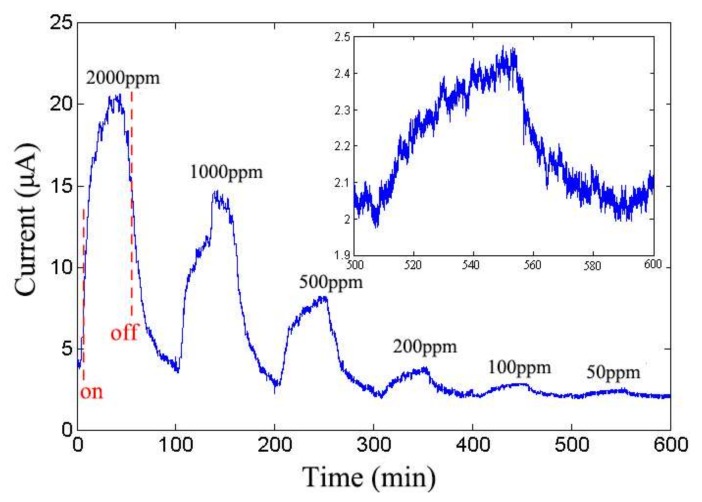
Evolution of nanodevice current with respect to the concentration of CO gas at 200 °C (inset is the zoom-in image when the CO concentration is 50 ppm).

The sensitivity of the ZnO sensor is measured by CO gas. The working temperature range from 100 °C to 450 °C. To avoid the failure of sensor at high temperature [35], the working temperature is set to 200 °C and the bias voltage is set to 0.3 V. The evolution of current with time is shown in Figure 6. 

**Figure 7:**
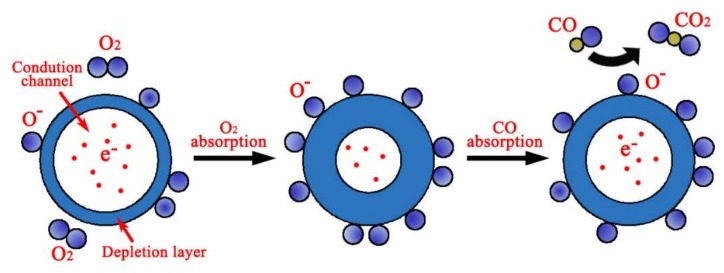
Schematics of ZnO CO sensor sensing mechanism.

“Turn on” and “Turn off” labels mean setting the target gas concentration as the determined concentration and zero, respectively. The rate of CO is 20 mL/min and ~50 min is needed to reach the target concentration or decreases from target concentration to zero. The CO sensing mechanism includes the desorption of adsorbed surface oxygen and grain boundaries in ZnO, exchange of charges between adsorbed CO molecule and the ZnO surface leading to changes in depletion depth [36], and changes in surface or grain boundary conduction by CO adsorption/desorption [37,38]. Figure 7 shows the schematics of ZnO CO sensor sensing mechanism. 

When ZnO is exposed to air atmosphere, oxygen molecules could be adsorbed on the ZnO surface and capture free electrons from them to form oxygen ions as described by

(4) $$O_{2}(g)+2e^{-}\to 2O^{-}(\mathrm{ads})$$

The equilibrium of the chemisorption process leads to the formation of depletion layer on the surface area and the resistance of the ZnO sensor is increased. When ZnO is exposed to the CO gas, these molecules react with the oxygen ions and release electrons back to ZnO, which increases the conductivity. The current of the device is increased at the same excitation voltage with respect to the increasing of CO concentration, which means that there exists the correlation between the current and concentration of CO. Although the sensitivity of the gas nanosensor is often defined by resistance [39,40,41,42] using commonly used formula, in this work, the current response of the sensor is transformed into a sensitivity value, which can be quantified by

(5) $$s=I_{k}/(I_{air}\times C)$$

where C and $I_{air}$ are the concentration of CO and the current in air, respectively. $I_{k}$ denotes the current when the concentration of CO is k. For example, $I_{500}$ denotes the current of the device when the tested CO is 500 ppm. It is clearly known from Equation (5) that there is a relationship between *s* and $I_{k}$ such that *s* is enhanced by increasing $I_{k}$. In Figure 7, $I_{air}$ is less than 2 μA and $I_{500}$ is ~8 μA, hence $s_{500}$ is 8 μA/(2 μA × 500 ppm) = 0.008 ppm^−1^. 

Before comparing the results obtained, it should be mentioned that Hsueh et al. [43] measured the performance of the sensor and the sensitivity of the device was defined by (*R_a_* − *R_b_*)/*R_a_* × 100%, where *R_a_* and *R_b_* are resistances in air and in CO gas, respectively. The results in Ref. [43] showed that the sensitivity of the device measured at 320 °C was around 57% when the concentration of CO was 500 ppm. With Equation (5), the value of sensitivity of misaligned ZnO CO sensor is changed to 0.00465 ppm^−1^. To compare the sensitivity of two CO sensors, a similar temperature with the Ref. [43] is set in the ensuing discussion.

**Figure 8:**
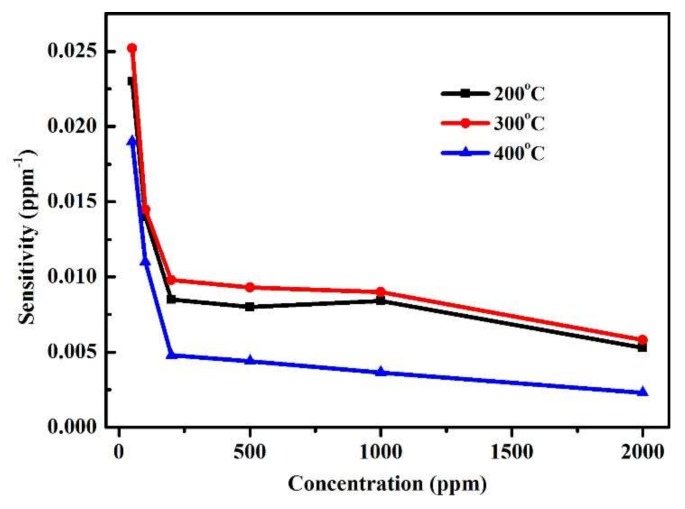
Relationship of sensitivity with respect to concentration of CO at different operating temperatures.

Figure 8 depicts the sensitivity of the device with respect to CO concentrations at different operating temperatures. It can be seen that the CO sensor is typically more sensitive to small concentration changes, and has high rates of response as the conduction path involves tunnelling through the depletion layer [31]. At each operating temperature, the sensitivity decreases when the CO gas concentration increases. The sensitivity does not exhibit significant differences when CO gas concentration is greater than 500 ppm as the carrier concentration inside the nanowire approaches saturation when the concentration of the reductive gas is increased [44]. It is well accepted that the sensitivity of semiconductor gas sensors is attributed to the chemisorption of oxidizing gases in the adsorption and the oxidation of reducing gases by previously chemisorbed oxygen at the surface of the metal oxide [45]. Takata et al. [46] found that the stable oxygen ions were O^−^ between 100 °C and 300 °C, and O^2−^ above 300 °C. The relevant reactions on the surface area are as follows

(6) $$\mathrm{CO}(g)+O^{-}(\mathrm{ads})\to {\mathrm{CO}}_{2}(g)+e^{-}$$

(7) $$\mathrm{CO}(g)+O^{2-}(\mathrm{ads})\to {\mathrm{CO}}_{2}(g)+2e^{-}$$

The molecular or atomic oxygen chemisorption captures a free electron from the conduction band giving adsorbed O^2−^ or O^−^, respectively. O_2_ is not reactive at all [47]. It is clear that the surface reaction in Equation (7) is more desirable for the gas sensing because the reaction could release more electrons to reduce the resistance of the nanowires and thus the gas sensitivity is increased. The surface reaction can be represented mainly by Equation (7) when the temperature is higher than 300 °C. The sensing mechanism at 300 °C may involve two types of reactions, as described by Equations (6) and (7), because, at the threshold temperature of 300 °C, the dominant oxygen species absorbed on the ZnO surface might be O^−^ and O^2−^. Furthermore, increase in CO response with increasing the operating temperature can be explained as the thermal energy is high enough to overcome the activation energy barrier to the reaction [48]. When the operating temperature is increased to 400 °C, the sensitivity of the nanodevice is decreased, possibly because these adsorbed oxygens start to desorbed from ZnO surface at this temperature. In addition, it is found that, when the concentration of CO is 500 ppm at 300 °C, the sensitivity of our sensor is 0.0093 ppm^−1^, which is higher than the result from Hsueh’s work at a similar temperature. This because the greater surface area of the aligned ZnO nanowires leads to stronger interactions between the adsorbed gases and the sensor surface, and thus the higher gas sensing sensitivity.

## 4. Conclusions

ZnO nanowires were orderly arranged on the silicon substrate using a common makeup brush. More than 80% of the nanowires are aligned within 20 degrees with respect to the combing direction. The I–V curve of the ZnO nanodevice is highly nonlinear and nearly symmetrical with respect to the applied voltage, which shows Schottky nature. The ultraviolet characteristics show nearly a 22-fold enhancement in response due to the photon adsorption and desorption of oxygen. The carbon monoxide (CO) sensing properties were tested at operating temperatures of 200, 300 and 400 °C. It was found that ZnO based sensor exhibited the highest sensitivity to CO at 300 °C due to the change of dominant oxygen species. The CO sensor is typically more sensitive to small concentration changes and the sensitivity does not exhibit significant differences when CO gas concentration is greater than 500 ppm because the carrier concentration inside the nanowire approaches saturation when the concentration of the reductive gas is increased. The CO sensing mechanism includes the desorption of adsorbed surface oxygen and grain boundaries in ZnO, exchange of charges between adsorbed CO molecule and the ZnO surface leading to changes in depletion depth. Moreover, the measurement results reveal that the sensor based on assembled ZnO nanowire has higher sensitivity than previous sensors. In conclusion, the nanowire assembly by combing process is easy to implement and a novel fabricating technique is provided for the nanodevice.
